# The Influence of Decisional and Emotional Forgiveness on Attributions

**DOI:** 10.3389/fpsyg.2019.01425

**Published:** 2019-06-25

**Authors:** Stephanie Lichtenfeld, Markus A. Maier, Vanessa L. Buechner, Maria Fernández Capo

**Affiliations:** ^1^School of Education, Durham University, Durham, United Kingdom; ^2^Department of Psychology, Ludwig-Maximilians-University of Munich, Munich, Germany; ^3^Department of Basic Sciences, Universitat Internacional de Catalunya, Barcelona, Spain

**Keywords:** emotional forgiveness, decisional forgiveness, attributions, forgiveness, emotion

## Abstract

Research on forgiveness suggests that forgiveness is an emotion-focused coping process important for clinical settings as it can promote both physical and mental health (Worthington et al., 2005; Witvliet and McCullough, 2007). Investigating antecedents of forgiveness, empirical studies and theoretical models propose that attributions influence forgiveness. However, hardly any studies or theoretical models have ever looked at the possibility that this relationship may be reciprocal in nature and whether forgiveness also impacts a victim’s attributions has not been investigated. The present, highly powered (*n* = 969) study seeks to fill this gap and provides the first empirical support that emotional forgiveness has a strong influence on subsequent attributions. Specifically, individuals, who have emotionally forgiven a transgression, hold the transgressor less responsible for the offense compared to those in the decisional forgiveness and control condition. Moreover, the findings conceptually replicate previous research (Lichtenfeld et al., 2015) by demonstrating that emotional, but not decisional forgiveness affects cognition and, thus, emotional and decisional forgiveness should be treated as distinct facets in the forgiveness process. Implications of these results for clinical and health psychology are discussed.

## Introduction

Much research has shown that forgiveness is linked to lower levels of anxiety and depression (Freedman and Enright, 1996; Rye and Pargament, 2002; Reed and Enright, 2006) and is also associated to benefits in both physical and mental health (Thoresen et al., 2000; Berry and Worthington, 2001; Witvliet et al., 2004). Furthermore, forgiveness has been found to increase the likelihood of restoring a social relationship (Tsang et al., 2006). Specifically, forgiveness serves as an important response in interpersonal interactions to “*maintain relatedness with fellow humans in the face of being harmed by them*” (Fincham et al., 2005). To maintain a positive relationship, it seems dysfunctional to hold the transgressor entirely responsible for an offense. Thus, forgiveness should lead to a change in causal attributions toward the offender.

While definitions of forgiveness differ substantially, Enright et al. (1992), and more recently Freedman and Zarifkar (2016), pointed out that most researchers agree that forgiveness is distinct from several other related constructs such as pardoning (a legal term for absolving a person of his/her guilt), condoning (which entails that the offense is justifiable), excusing (which entails that there are mitigating factors that led to the offense), forgetting (which suggests that the offense is not consciously accessible), or reconciliation (which involves the restoration of the relationship; McCullough and Witvliet, 2002). Likewise, as posited by Goertzen (2003, p. 4), forgiveness also differs from defense mechanisms such as denial, suppression, repression, or dissociation, because “they involve a refusal to acknowledge the offense.”

Forgiveness is linked to physical and mental health and thus plays an important role in the clinical context. In respect to psychological benefits, forgiveness has been found to be associated with a reduction in negative emotions. For instance, Coyle and Enright found that a forgiveness intervention reduced anxiety, anger, and grief in post-abortion men (Coyle and Enright, 1997). Likewise, Freedman and Enright (1996) found that anxiety and depression decreased in incest survivors when taking part in a forgiveness intervention. A study with substance dependent patients also revealed that forgiveness therapy led to decreases in anger, anxiety, and depression (Lin et al., 2004). When comparing forgiveness therapy to an alternative therapy (including anger validation with mourning, assertiveness strategies, and interpersonal skills) emotionally abused women experienced greater improvement in their depression, trait anxiety, and post-traumatic stress symptoms in the forgiveness therapy group (Reed and Enright, 2006). Moreover, forgiveness mediated the relationship between interpersonal violence and post-traumatic stress disorder [PTSD; (Orcutt et al., 2005)].

Forgiveness has also been shown to have physiological benefits. It is associated to less physiological stress responses and superior health outcomes, while not forgiving can raise skin conductance, heart rate, and blood pressure (Toussaint et al., 2016). A study by Carson et al. (2005) further yielded that emotions mediate the relationship between forgiveness and chronic low back pain. Specifically, individuals, who suffered from chronic low back pain, experienced lower levels of sensory pain when they were more forgiving and state anger mediated the relationship between forgiveness and sensory pain. Finally, forgiveness has social benefits increasing the likelihood of restoring the relationship (Raj and Wiltermuth, 2016). Particularly, it induces prosocial feelings both in the victim (McCullough et al., 1997; McCullough, 2000; Worthington, 2006) and in the transgressor (Kelln and Ellard, 1999; Mooney et al., 2016).

In sum, forgiveness is associated with less negative and more positive aspects of physical and mental health and, specifically, the emotional processes involved seem to play an important role in this relationship.

When looking at the process of forgiveness a considerable amount of research has investigated the relationship between attributions and forgiveness and found that attributions are indeed linked to forgiveness. Based on attribution theory and the theory of correspondent interference (Heider, 1958; Jones and Davis, 1965; Shaver, 1985; Weiner, 1985, 1995), Bradfield and Aquino (1999) could show that, in the workplace, attributions of blame were positively related to revenge cognitions and negatively related to forgiveness cognitions. Both revenge and forgiveness cognitions were, in turn, linked to revenge and forgiveness behavior. Davis and Gold (2011) found that apologies lead a victim to perceive the offending behavior as unlikely to occur again, causing decreases in attributions of behavioral stability and thus eliciting forgiveness [see also the models on attribution; (Weiner et al., 1991; Gold and Weiner, 2000; Takaku, 2001; Gold and Davis, 2005)]. In a study on domestic violence, Gordon et al. (2004) discovered that women, who appraise their partner’s behavior as less malicious and intentional, are more likely to forgive the behavior and to consider continuing the relationship. In a similar vein, Hall and Fincham (2006) found that conflict-promoting attributions of individuals (i.e., internal, global, and stable attributions), who had experienced infidelity, may inhibit forgiveness processes. They suggest that this may make couples more susceptible to negative behaviors (i.e., avoidance and revenge), thereby increasing the likelihood of relationship dissolution. In a longitudinal study, McCullough et al. (2003) revealed that during periods when participants experienced more empathy and made fewer responsibility attributions than usual, they experienced more forgiveness as indicated by lower levels of avoidance motivation, revenge motivation, and higher levels of benevolence motivation than usual.

An experimental study (Takaku, 2006), which was based on the dissonance-attribution model of interpersonal forgiveness (Takaku, 2001; Takaku et al., 2001), yielded that individuals in the hypocrisy-induced dissonance condition (i.e., by making them aware of their own past wrongdoing) perceived the cause of a reckless driver’s misbehavior as less internal and less stable, experienced significantly less negative emotional reaction, and showed more intention to forgive and less negative behavioral intention than participants in the non-dissonance condition.

In his conceptual analysis of forgiveness, Fincham (2000) likewise suggests that attributed responsibility influences the degree of forgiveness, which in turn causes less retaliatory and more reconciliatory relationship behavior of spouses. Individuals, who hold their spouses less accountable for a harm-doing event, are more likely to forgive their spouses than individuals who attribute more responsibility to their spouses (Fincham, 2000). Thus, forgivers apparently are inclined to give their transgressors “the benefit of the doubt” (McCullough, 2001). In line with that, Fincham et al. (2002) found that positive relationship quality was associated with more benign causal and responsibility attributions, which in turn facilitated forgiveness.

In sum, both empirical and theoretical work suggests a relationship between attributions and forgiveness. However, empirical studies and theoretical models predominantly suggest a causal direction of attributions affecting forgiveness. Hardly any studies or theoretical models have ever looked at the possibility that this relationship may be reciprocal in nature and investigated if forgiveness has an impact on a victim’s attributions. One study did investigate the effect of forgiveness on attributions by running a longitudinal study (Wenzel et al., 2010). However, in this study only the effect of forgiveness on attributions of severity, but not on responsibility attributions, were found. This may be due to the fact that the sample size (*n* = 112) was rather low to run multivariate analyses and to detect stable correlations [for a discussion on stability of correlations, see also Schönbrodt and Perugini (2013)]. Moreover, a longitudinal design, while being more informative than a cross-sectional design, can still not establish a causal relationship. Thus, the current study aims at investigating the causal effect of forgiveness on appraisals by running an experimental design in a highly powered study. In general, research on the consequences of forgiveness is still sparse (Karremans and Van Lange, 2008). This is specifically the case for experimental research allowing for causal interpretations of cognitive, affective, and behavioral consequences of forgiveness. Nevertheless, it seems an important question if not only forgiveness is more easily granted when the offender is held less responsible for a transgression, but also if forgiveness itself has consequences on one’s cognitions, such as a victim’s attributions regarding the offender. In our view, it seems very plausible that forgiveness influences a victim’s attributions about the offender. If a person forgives a transgressor it seems adaptive to change one’s attributions about the event accordingly and serve the evolutionary goal of forgiveness to reestablish the relationship.

Another important aspect is that research on forgiveness and attributions looked at forgiveness merely as a global construct and did not differentiate between different types of forgiveness. However, several researchers have highlighted different facets of the forgiveness process. A meta-analysis by Strelan and Covic (2006) shows that several process models of forgiveness incorporate both the decision to forgive (Fitzgibbons, 1986; Enright, 1996; Worthington, 1998, 2001) as well as an understanding of, or empathy for, the offender (Fitzgibbons, 1986; Enright, 1996; Gordon and Baucom, 1998; Worthington, 1998, 2001; Malcolm and Greenberg, 2000). Worthington et al. (2007) emphasize this differentiation by distinguishing between decisional and emotional forgiveness [e.g., (Davis et al., 2015)], which they consider to be related but distinct processes of forgiveness. Decisional forgiveness is defined as the behavioral intention statement that one seeks to reduce one’s negative behavior and (if possible and appropriate) restore positive behavior toward the offender. Nevertheless, even when making a sincere decision to forgive, one may still feel emotionally unforgiving (e.g., angry, resentful, and hurt) toward the offender. Emotional forgiveness, in turn, is considered as the replacement of negative emotions with positive ones [e.g., empathy, love, compassion; see Hook et al. (2012); for a review of empirical evidence in support of this distinction see Worthington, 2006]. Given that emotional forgiveness leads to more positive and less negative feelings, it should likewise lead to more positive attributions concerning the transgressor.

In line with the proposition by Worthington et al. (2007) that decisional forgiveness differs substantially from emotional forgiveness, Lichtenfeld et al. (2015) show that emotional and decisional forgiveness are indeed distinct subcomponents of forgiveness and, as a consequence, differentially influence cognitive processes. In their study, emotional and decisional forgiveness were experimentally manipulated and the results yielded a positive effect of emotional forgiveness on the subsequent forgetting of offense relevant traits. One plausible mechanism explaining this positive effect of emotional forgiveness on forgetting is that the offender is held less responsible for a transgression and, as a result, offense relevant traits are less accessible for the victim and are thus forgotten. In line with that, we expected emotional forgiveness to cause decreases in responsibility judgments of an offender.

In sum, the aim of the present study was to investigate the causal effects of emotional and decisional forgiveness on responsibility attributions. We hypothesized that emotional forgiveness would lead to significantly less responsibility attributions in respect to the transgressor. Moreover, we sought to conceptually replicate the findings from Lichtenfeld et al. (2015) paper with a high powered sample and show that emotional and decisional forgiveness are indeed two separate facets, which differentially influence cognitive processes.

## Materials and Methods

In line with Simmons et al. (2012) proposal for a 21 word solution of disclosure, we report how we determined our sample size, all data exclusions (if any), all manipulations, and all measures in the study in the following section.

### Ethics Statement

The research reported herein was conducted at the LMU Munich and was approved by the ethics committee of the Department of Psychology, LMU Munich, in accordance with the ethical standards expressed in the Declaration of Helsinki. All participants gave written informed consent and were thoroughly debriefed. The individuals’ written consent was obtained after reading the instruction to the experiments. The experimenter emphasized that they will receive their credit also if they decided not to participate in this study. Participants were also told that they could stop and leave the experiment at any point of time. This consent procedure has been approved by the ethics committee.

### Participants

Before the beginning of the study, an *a priori* power analysis suggested a sample size of 969 participants to provide 80% power at α = 0.05 assuming a small effect size (*d* = 0.20) given a between subject design with three separate experimental groups. Thus, a total of 969 participants (626 female, *mean age* = 23.64 years, and *SD* = 4.25) at a German university took part for credit.^1^ (Allemand, 2008) Participants were tested individually or in small groups seated in individual cubicles and responding to a paper-pencil format questionnaire.

### Materials

#### Responsibility Judgments

Adapted from Struthers et al. (2010) and based on Weiner’s (1995) conceptualization of responsibility inferences, judgments of responsibility were measured using three composite causal dimensions: locus of causality, controllability, and responsibility. Unless otherwise indicated, the items were rated on a seven-point scale ranging from 1 (not at all) to 7 (very much so).

Locus of causality was measured using two items: “Would you say that the main cause of the event … ?” which was rated on a seven-point scale ranging from 1 (reflected an aspect of the situation) to 7 (reflected an aspect of your fellow student); and “To what extent do you think the cause of the event had something to do with your fellow student?” Controllability was also measured using two items: “Was the main cause of the event something that was … ?” which was rated on a seven-point scale ranging from 1 (not controllable by your fellow student) to 7 (controllable by your fellow student); and “To what extent do you think your fellow student had control over the negative event?” Responsibility was also measured using two items: “How responsible was your fellow student for the event?”; and “How accountable do you think your fellow student was for the event?”

### Procedure

At the beginning of the experiment, participants were provided with a description and illustration of a scenario. In accord with Worthington et al.’s (2007) call for ecological validity that studies must employ tasks that mirror daily life, we used a scenario that was a realistic offense experienced by students at a university [see Lichtenfeld et al. (2015)]. Participation was restricted to participants between 18 and 40 years of age, so that the described scenario was indeed a situation that may have been encountered by participants within the past few years.

The scenario described a situation in which a group of students had to prepare a presentation for a class in which the individual has a particularly innovative idea as to how to run the presentation. When telling the professor about the plan and the professor likes it, another student, the transgressor, proclaims the idea to be hers and consequently gets a better grade than the rest of the group.

After reading the scenario, participants were randomly assigned to one of three between-subject conditions: the decisional forgiveness condition, the emotional forgiveness condition, or the control condition. Forgiveness was manipulated using an adapted version of Worthington et al.’s (2007) manipulation procedure, which has been used in previous research (Lichtenfeld et al., 2015). Specifically, participants in the decisional forgiveness condition were asked to “think of the offender as a “human being” who behaved badly. Even if the relationship cannot be restored, try to resolve not to take revenge on the person, but to behave positively and not negatively toward the offender.” Participants in the emotional forgiveness condition were asked to “think of the offender as a “human being” who behaved badly. Even if the relationship cannot be restored, try to genuinely wish that the offender experiences something positive or healing. Even though it may be hard, focus your thoughts and feelings on giving a gift of empathy or compassion.” Those in the control group, in turn, were asked to think about one’s own thoughts, feelings, and bodily reactions in this situation; “What would you think? How would you feel? How would your body react? What would you do in such a situation? Think of all aspects that would be influenced in such a situation?” Then, participants were asked to answer three filler questions related to the scenario, and the appraisal questions in respect to the offender.

Finally, participants answered questions that were not related to the study at hand and completed a brief demographics questionnaire, given their extra credit, were debriefed, and dismissed.

## Results

### Composite Variables

In line with Struthers et al. (2010) paper and based on positive inter-item correlations and acceptable levels of internal consistency, a composite scale was created for responsibility attributions (*M* = 5.59, *SD* = 0.95; α = 0.81).

### Effects of Forgiveness Induction

**FIGURE 1:**
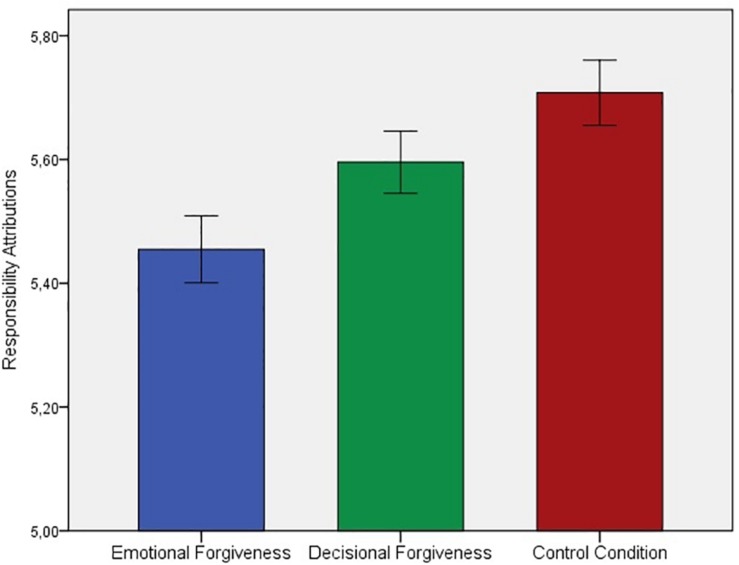
Responsibility attributions as a function of forgiveness manipulation. Standard errors are indicated by vertical lines.

Conducting an analysis of variance (ANOVA), we found a significant effect of condition on the composite responsibility scale [see Struthers et al. (2010)], *F*(2,966) = 5.84, *p* = 0.003, η_p_^2^ = 0.012, indicating that participants differed in their overall responsibility appraisals ascribed to the transgressor (see Figure 1). *A priori* analyses were directed and thus tested one-tailed. The analyses yielded that participants in the emotional forgiveness condition judged the offender as less responsible (*M* = 5.45, *SD* = 0.97) than those in the decisional forgiveness (*M* = 5.60, *SD* = 0.91), *t*(645) = −1.90, *p* = 0.029, and *d* = −0.15 (one-tailed), and were holding the transgressor less responsible for the offense than those in the control condition (*M* = 5.71, *SD* = 0.94), *t*(643) = 3.35, *p* < 0.001, and *d* = −0.26 (one-tailed). Whereas, participants in the decisional forgiveness condition tended to differ from those in the control condition in respect to their controllability appraisals ascribed to the transgressor, *t*(644) = −1.54, *p* = 0.062, and *d* = −0.12 (one-tailed). Given that gender has been found to be a significant predictor of forgiveness with females being more forgiving than males (Miller et al., 2008), we further tested if the results hold when controlling for participants’ gender. Thus, an analysis of covariance (ANCOVA) was conducted including gender as a covariate. No effect of gender on responsibility attributions could be found, *F*(1,921) = 0.210, *p* = 0.647, η_p_^2^ < 0.001 and the results further support our findings by yielding almost identical results for the effect of forgiveness on attributions, *F*(2,921) = 5.571, *p* = 0.004, η_p_^2^ = 0.012.

## Discussion

The results of the experiment reported herein provide initial evidence for the effects of forgiveness on responsibility attributions. In line with our propositions, the findings of the present study indicate that forgiveness causes lower levels of responsibility attributions. More specifically, individuals, who were asked to think about emotionally forgiving a transgressor, held the transgressor less responsible for the offense, judged the situation as less controllable by the offender and appraised the main cause of the event being more due to situational factors than those who do not forgive. While participants in the decisional forgiveness condition likewise tended to obtain lower ratings on the various attribution scales, they did not significantly differ from those in the control condition. In sum, the present work extends existing empirical work and theoretical models on forgiveness by demonstrating a causal relation of forgiveness on responsibility attributions. Moreover, the findings support previous theory (Worthington et al., 2007) and research (Lichtenfeld et al., 2015) by showing that only individuals, who are asked to forgive emotionally, substantially differ in respect to their cognitive processes (e.g., their responsibility attributions) compared to those in the decisional forgiveness and the control condition, and thus a differentiation between emotional and decisional forgiveness seems necessary.

This finding is also in line with emotional focused therapy, which suggests that emotions are fundamental aspects in the construction of the self and are central to determining self-organization (Greenberg, 2004). Given that emotions are an adaptive form of information-processing and action readiness, which orient people to their environment and promote their well-being (Frijda, 1986; Greenberg and Safran, 1987; Greenberg and Paivio, 1997), emotional focused therapy aims to enhance emotion-focused coping by supporting people to become aware of, accept, and make sense of their emotional experience (Greenberg, 2004). Thus, the fact that emotional forgiveness is driving the effect on attributions supports the notion that emotions are at the core of changing behavior, cognition, and feelings in interventions and therapies.

Evolutionary theorizing (McCullough, 2008) suggests that forgiveness serves the function of restoring beneficial relationships after experiencing an interpersonal harm. To be able to re-establish a positive relationship with an offender and change one’s attitude toward them seems adaptive. Individuals, who have been hurt, are inclined to make responsibility attributions about the offense. For instance, when placed in a victim role, individuals are more likely to portray offenses as harmful, intentional, and malicious (Exline et al., 1998). But, when trying to restore a valuable relationship it seems counterproductive to hold the transgressor entirely responsible for the offense, view the situation as fully controllable by the offender, and appraise the main cause of the event as being predominantly due to personal factors of the offender. The present results support this theorizing by showing that the extent to which an offender is held responsible for a transgression is changed when individuals emotionally forgive an offender. While previous empirical studies and theoretical models predominantly suggest a causal direction of attributions affecting forgiveness, the present study suggests that forgiveness has a causal impact on individuals’ attributions. Nevertheless, it is possible that not only forgiveness influences attributions, but that this relationship is reciprocal in nature, which may even result in a virtuous circle with forgiveness fostering more benevolent attributions about the offender, which in turn leads the victim to take a more forgiving stance toward the offender.

While forgiveness has been shown to significantly improve health and wellbeing in many interventions and therapies (Freedman and Enright, 1996; Coyle and Enright, 1997; Lin et al., 2004; Carson et al., 2005; Orcutt et al., 2005; Reed and Enright, 2006; Raj and Wiltermuth, 2016), and the positive effect of forgiveness on attributions could pave the way for further steps in the restoration of relationships, some authors have questioned the benefits of forgiveness by mainly arguing that forgiveness enhances abuse and repetition of transgressions (Gordon et al., 2004; Wallace et al., 2008; Luchies et al., 2010; McNulty, 2011). For instance, Gordon et al. (2004) reported that women living in domestic violence shelters were more likely to form the intention to return to their partners when they experience more forgiveness and thus state that forgiveness may have detrimental consequences in this instance. Nevertheless, recent research (McNulty, 2010) suggests that forgiveness is neither a panacea nor the devil’s work but that the relationship between forgiveness and negative outcomes is moderated by important contextual variables. Specifically, McNulty (2008) found that forgiveness was only related to less relationship satisfaction and more serious problems when partners showed negative behaviors more frequently, but forgiveness was related to greater relationship satisfaction and fewer problems in less troubled relationships. In a similar vein, forgiveness yielded a negative relationship with self-respect when partners were disagreeable or did not make amends, but forgiveness was related to more self-respect when partners were agreeable or made amends (Luchies et al., 2010). Thus, while the present study suggests that forgiveness positively impacts individuals’ attributions toward the transgressor, the question as to whether forgiveness serves a positive overarching outcome seems to be highly dependent on the severity as well as the context in which the transgression takes place. In line with these contextual considerations and discussions in regard to the severity of a transgression, the present study investigated the effect of emotional and decisional forgiveness on individuals’ changes in attributions when facing a realistic offense experienced in the university setting. This scenario was chosen to address the call for realistic scenarios (Worthington et al., 2007) to establish ecological validity. However, it is open to question whether more severe offenses will likewise cause changes in individuals’ attributions and, as discussed above, if forgiveness in such situations does indeed have beneficial consequences for the individual.

In addition, while ecological validity is enhanced by representing a realistic offense, its ecological validity is restricted by using a hypothetical scenario. While several studies in the literature on forgiveness and attributions find that both naturalistic and scenario based experiments reveal similar findings [e.g., (Struthers et al., 2010)], it is open to question if the current results, that emotional forgiveness causes changes in individuals’ attributions of the transgressor, will replicate in a naturalistic setting. Moreover, the description of the scenario was discussing an offense of a fellow student, which could potentially be a friend but does not need to be one. However, this was not stated explicitly and, thus, there may have been some variation in perceived closeness toward the transgressor. Given that a victim’s willingness to forgive has repeatedly been shown to be influenced by the closeness of the relationship between victim and offender, and victims have been found to be generally more inclined to forgive those they hold a close relationship with (i.e., McCullough et al., 1998; Karremans and Aarts, 2007), this might influence the likeliness to forgive depending on the type of relationship individuals were thinking about. However, given the large sample size it seems unlikely that there were differences in the average perception of relationship closeness between groups. Nevertheless, further studies should investigate the effects of forgiveness on attributions in more naturalistic settings and focus on the kind of relationships held with the transgressor to test for the generalisability of findings, as well as to investigate differences in attributions as a result of the forgiveness process depending on the type of relationship someone holds with the transgressor.

Changing one’s attributional cognitions about an event has important implications for the relationships and the fact that forgiveness leads individuals to readjust their judgment on a person makes restoration of closeness more likely. This may also be incorporated in forgiveness interventions. For instance, in his REACH Forgiveness Model, which is designed to assist individuals in the forgiveness process, Worthington (2006) proposes five steps to REACH emotional forgiveness. After the offended person recalls (R) the event without blaming the offender or self-pitying, he makes a conscious attempt to decide to forgive and attempts to empathize (E) with the offender and seeks to replace negative emotional states with positive ones. The altruistic gift of forgiving (A) incorporates the person’s decision to forgive and their emotional experience. To strengthen the decision to forgive and the experience of emotional forgiveness the individual is asked to make a public commitment (C). This commitment is intended to help the person hold onto forgiveness (H). The fact that forgiveness leads to changes in responsibility attributions may have a positive influence on people and help them in holding on to their decision to forgive.

In sum, two important messages can be derived from the present research. First, the study results suggest that emotional forgiveness has an effect on individuals’ attributional patterns and, thus, the causal link between forgiveness and attributions is at least reciprocal in nature and should thereby be considered in future theory and research. Second, the results imply that emotional and decisional forgiveness are two distinct facets in the forgiveness process and should be handled as such in future theorizing and research.

## Ethics Statement

The research reported herein was conducted at the LMU Munich and was approved by the ethics committee of the Department of Psychology, LMU Munich, in accordance with the ethical standards expressed in the Declaration of Helsinki. All participants gave written informed consent and were thoroughly debriefed. The individuals’ written consent was obtained after reading the instruction to the experiments. The experimenter emphasized that they will receive their credit also if they decided not to participate in this study. Participants were also told that they could stop and leave the experiment at any point of time. This consent procedure has been approved by the ethics committee.

## Author Contributions

SL, MM, and VB developed the study concept and contributed to the study design. SL performed the testing and data collection. SL, MM, and VB performed the data analysis and interpretation. SL drafted the manuscript. MM, VB, and MF provided the critical revisions. All authors approved the final version of the manuscript for submission.

## Conflict of Interest Statement

The authors declare that the research was conducted in the absence of any commercial or financial relationships that could be construed as a potential conflict of interest.

## Footnotes

1 Given that age has been shown to be a significant predictor of forgiveness (Allemand, 2008), we restricted the sample to participants under 40 years of age. The 7 participants, who were older than 40, were *a priori* excluded from the sample. The magnitude and significance levels of all reported results remained the same with these participants included.

## References

[B1] Allemand M. (2008). Age differences in forgivingness: the role of future time perspective. *J. Res. Pers.* 42 1137–1147. 10.1016/j.jrp.2008.02.009

[B2] Berry J. W. Worthington E. L. (2001). Forgivingness, relationship quality, stress while imagining relationship events, and physical and mental health. *J. Couns .Psychol.* 48 447–455. 10.1037/0022-0167.48.4.447

[B3] Bradfield M. Aquino K. (1999). The effects of blame attributions and offender likeableness on forgiveness and revenge in the workplace. *J. Manage.* 25 607–631. 10.1177/014920639902500501

[B4] Carson J. W. Keefe F. J. Goli V. Fras A. M. Lynch T. R. Thorp S. R. (2005). Forgiveness and chronic low back pain: a preliminary study examining the relationship of forgiveness to pain, anger, and psychological distress. *J. Pain* 6 84–91. 10.1016/j.jpain.2004.10.012 15694874

[B5] Coyle C. T. Enright R. D. (1997). Forgiveness intervention with postabortion men. *J. Consult. Clin. Psychol.* 65 1042–1046. 10.1037//0022-006X.65.6.1042 9420366

[B6] Davis D. E. Hook J. N. Van Tongeren D. R. DeBlaere C. Rice K. G. Worthington E. L. Jr. (2015). Making a decision to forgive. *J. Couns. Psychol.* 62 280–288. 10.1037/cou0000054 25621589

[B7] Davis J. R. Gold G. J. (2011). An examination of emotional empathy, attributions of stability, and the link between perceived remorse and forgiveness. *Pers. Individ. Dif.* 50 392–397. 10.1016/j.paid.2010.10.031

[B8] Enright R. D. Gassin E. A. Wu C. R. (1992). Forgiveness: a developmental view. *J. Moral Educ.* 21 99–114. 10.1080/0305724920210202

[B9] Enright R. D. (1996). Counseling within the forgiveness triad: on forgiving, receiving forgiveness, and self-forgiveness. *Couns. Values* 40 107–126. 10.1002/j.2161-007X.1996.tb00844.x

[B10] Exline J. J. Yali A. M. Lobel M. (1998). “Self-serving perceptions in victim and perpetrator accounts of transgressions,” in *Proceedings of the Annual Meeting of the Midwestern Psychological Association*, Chicago, IL.

[B11] Fincham F. D. (2000). The kiss of the porcupines: from attributing responsibility to forgiving. *Pers. Relatsh.* 7 1–23. 10.1111/j.1475-6811.2000.tb00001.x

[B12] Fincham F. D. Jackson H. Beach S. R. H. (2005). Transgression severity and forgiveness: different moderators for objective and subjective severity. *J. Soc. Clin. Psychol.* 24 860–875. 10.1521/jscp.2005.24.6.860

[B13] Fincham F. D. Paleari F. G. Regalia C. (2002). Forgiveness in marriage: the role of relationship quality, attributions, and empathy. *Pers. Relatsh.* 9 27–37. 10.1111/1475-6811.00002

[B14] Fitzgibbons R. P. (1986). The cognitive and emotive uses of forgiveness in the treatment of anger. *Psychother. Theor. Res. Pract. Train.* 23 629–633. 10.1037/h0085667

[B15] Freedman S. Zarifkar T. (2016). The psychology of interpersonal forgiveness and guidelines for forgiveness therapy: what therapists need to know to help their clients forgive. *Spiritual. Clin. Pract.* 3 45–58. 10.1037/scp0000087

[B16] Freedman S. R. Enright R. D. (1996). Forgiveness as an intervention goal with incest survivors. *J. Consult. Clin. Psychol.* 64 983–992. 10.1037/0022-006X.64.5.983 8916627

[B17] Frijda N. H. (1986). *The Emotions.* Cambridge: Cambridge University Press.

[B18] Goertzen L. R. (2003). *Conceptualizing Forgiveness within the Context of a Reversal Theory Framework: The Role of Personality, Motivation, and Emotion.* Windsor: University of Windsor.

[B19] Gold G. J. Davis J. R. (2005). Psychological determinants of forgiveness: an evolutionary perspective. *Humboldt J. Soc. Relat.* 29 111–134. 10.2307/23262798

[B20] Gold G. J. Weiner B. (2000). Remorse, confession, group identity, and expectancies about repeating a transgression. *Basic Appl. Soc. Psychol.* 22 291–300. 10.1207/15324830051035992

[B21] Gordon K. C. Baucom D. H. (1998). Understanding betrayals in marriage: a synthesized model of forgiveness. *Fam. Process.* 37 425–449. 10.1111/j.1545-5300.1998.00425.x 9934566

[B22] Gordon K. C. Burton S. Porter L. (2004). Predicting the intentions of women in domestic violence shelters to return to partners: does forgiveness play a role? *J. Fam. Psychol.* 18 331–338. 10.1037/0893-3200.18.2.331 15222840

[B23] Greenberg L. S. (2004). Emotion–focused therapy. *Clin. Psychol. Psychother.* 11 3–16. 10.1002/cpp.388 19639649

[B24] Greenberg L. S. Paivio S. C. (1997). *Working with Emotions in Psychotherapy.* New York, NY: Guilford Press.

[B25] Greenberg L. S. Safran J. D. (1987). *Emotion in Psychotherapy: Affect, Cognition, and the Process of Change.* New York, NY: Guilford Press.

[B26] Hall J. H. Fincham F. D. (2006). Relationship dissolution following infidelity: the roles of attributions and forgiveness. *J. Soc. Clin. Psychol.* 25 508–522. 10.1521/jscp.2006.25.5.508

[B27] Heider F. (1958). *The Psychology of Interpersonal Relations.* New York, NY: Wiley.

[B28] Hook J. N. Worthington E. L. Jr. Utsey S. O. Davis D. E. Burnette J. L. (2012). Collectivistic self-construal and forgiveness. *Couns. Values* 57 109–124. 10.1002/j.2161-007X.2012.00012.x

[B29] Jones E. E. Davis K. E. (1965). “From acts to dispositions: the attribution process in person perception,” in *Advances in Experimental Social Psychology*, ed. Berkowitz L. (San Diego, CA: Academic Press), 220–266.

[B30] Karremans J. C. Aarts H. (2007). The role of automaticity in determining the inclination to forgive close others. *J. Exp. Soc. Psychol.* 43 902–917. 10.1016/j.jesp.2006.10.012

[B31] Karremans J. C. Van Lange P. A. M. (2008). Forgiveness in personal relationships: its malleability and powerful consequences. *Eur. Rev. Soc. Psychol.* 19 202–241. 10.1080/10463280802402609

[B32] Kelln B. R. C. Ellard J. H. (1999). An equity theory analysis of the impact of forgiveness and retribution on transgressor compliance. *Pers. Soc. Psychol. Bull.* 25 864–872. 10.1177/0146167299025007008

[B33] Lichtenfeld S. Buechner V. L. Maier M. A. Fernández-Capo M. (2015). Forgive and forget: differences between decisional and emotional forgiveness. *PLoS One* 10:e0125561. 10.1371/journal.pone.0125561 25946090 PMC4422736

[B34] Lin W.-F. Mack D. Enright R. D. Krahn D. Baskin T. W. (2004). Effects of forgiveness therapy on anger, mood, and vulnerability to substance use among inpatient substance-dependent clients. *J. Consult. Clin. Psychol.* 72 1114–1121. 10.1037/0022-006X.72.6.1114 15612857

[B35] Luchies L. B. Finkel E. J. McNulty J. K. Kumashiro M. (2010). The doormat effect: when forgiving erodes self-respect and self-concept clarity. *J. Pers. Soc. Psychol.* 98 734–749. 10.1037/a0017838 20438221

[B36] Malcolm W. M. Greenberg L. S. (2000). “Forgiveness as a process of change in individual psychotherapy,” in *Forgiveness: Theory, Research, and Practice*, eds McCullough M. E. Pargament K. I. Thoresen C. E. (New York, NY: Guildford Press), 179–202.

[B37] McCullough M. E. (2000). Forgiveness as human strength: theory, measurement, and links to well-being. *J. Soc. Clin. Psychol.* 19 43–55. 10.1521/jscp.2000.19.1.43

[B38] McCullough M. E. (2001). Forgiveness: Who does it and how do they do it? *Curr. Dir. Psychol. Sci.* 10 194–197. 10.1111/1467-8721.00147

[B39] McCullough M. E. (2008). *Beyond Revenge: The Evolution of the Forgiveness Instinct.* San Francisco, CA: Jossey-Bass.

[B40] McCullough M. E. Fincham F. D. Tsang J.-A. (2003). Forgiveness, forbearance, and time: the temporal unfolding of transgression-related interpersonal motivations. *J. Pers. Soc. Psychol.* 84 540–557. 10.1037/0022-3514.84.3.540 12635915

[B41] McCullough M. E. Rachal K. C. Sandage S. J. Worthington E. L. Jr. Brown S. W. Hight T. L. (1998). Interpersonal forgiving in close relationships: II. Theoretical elaboration and measurement. *J. Pers. Soc. Psychol.* 75 1586–1603. 10.1037/0022-3514.75.6.1586 9914668

[B42] McCullough M. E. Witvliet C. V. O. (2002). “The psychology of forgiveness,” in *Handbook of Positive Psychology*, eds Snyder C. R. Lopez S. J. (New York, NY: Oxford University Press), 446–458.

[B43] McCullough M. E. Worthington E. L. Jr. Rachal K. C. (1997). Interpersonal forgiving in close relationships. *J. Pers. Soc. Psychol.* 73 321–336. 10.1037/0022-3514.73.2.321 9248052

[B44] McNulty J. K. (2008). Forgiveness in marriage: putting the benefits into context. *J. Fam. Psychol.* 22 171–175. 10.1037/0893-3200.22.1.171 18266545

[B45] McNulty J. K. (2010). When positive processes hurt relationships. *Curr. Dir. Psychol. Sci.* 19 167–171. 10.1177/0963721410370298 24791710

[B46] McNulty J. K. (2011). The dark side of forgiveness: the tendency to forgive predicts continued psychological and physical aggression in marriage. *Pers. Soc. Psychol. Bull.* 37 770–783. 10.1177/0146167211407077 21558557 PMC4112745

[B47] Miller A. J. Worthington E. L. Jr. McDaniel M. A. (2008). Gender and forgiveness: a meta-analytic review and research agenda. *J. Soc. Clin. Psychol.* 27 843–876. 10.1521/jscp.2008.27.8.843

[B48] Mooney L. Strelan P. McKee I. (2016). How forgiveness promotes offender pro-relational intentions: the mediating role of offender gratitude. *Br. J. Soc. Psychol.* 55 44–64. 10.1111/bjso.12120 26150176

[B49] Orcutt H. K. Pickett S. M. Pope E. B. (2005). Experiential avoidance and forgiveness as mediators in the relation between traumatic interpersonal events and posttraumatic stress disorder symptoms. *J. Soc. Clin. Psychol.* 24 1003–1029. 10.1521/jscp.2005.24.7.1003

[B50] Raj M. Wiltermuth S. S. (2016). Barriers to forgiveness. *Soc. Pers. Psychol. Compass* 10 679–690. 10.1111/spc3.12290

[B51] Reed G. L. Enright R. D. (2006). The effects of forgiveness therapy on depression, anxiety, and posttraumatic stress for women after spousal emotional abuse. *J. Consult. Clin. Psychol.* 74 920–929. 10.1037/0022-006x.74.5.920 17032096

[B52] Rye M. S. Pargament K. I. (2002). Forgiveness and romantic relationships in college: Can it heal the wounded heart? *J. Clin. Psychol.* 58 419–441. 10.1002/jclp.1153 11920695

[B53] Schönbrodt F. D. Perugini M. (2013). At what sample size do correlations stabilize? *J. Res. Pers.* 47 609–612. 10.1016/j.jrp.2013.05.009

[B54] Shaver K. G. (1985). *The Attribution of Blame: Causality, Responsibility, and Blameworthiness.* New York, NY: Springer-Verlag.

[B55] Simmons J. P. Nelson L. D. Simonsohn U. (2012). A 21 word solution. *Dialogue* 26, 4–7.

[B56] Strelan P. Covic T. (2006). A review of forgiveness process models and a coping framework to guide future research. *J. Soc. Clin. Psychol.* 25 1059–1085. 10.1521/jscp.2006.25.10.1059

[B57] Struthers C. W. Eaton J. Mendoza R. Santelli A. G. Shirvani N. (2010). Interrelationship among injured parties’ attributions of responsibility, appraisal of appropriateness to forgive the transgressor, forgiveness, and repentance. *J. Appl. Soc. Psychol.* 40 970–1002. 10.1111/j.1559-1816.2010.00607.x

[B58] Takaku S. (2001). The effects of apology and perspective taking on interpersonal forgiveness: a dissonance-attribution model of interpersonal forgiveness. *J. Soc. Psychol.* 141 494–508. 10.1080/00224540109600567 11577848

[B59] Takaku S. (2006). Reducing road rage: an application of the dissonance-attribution model of interpersonal forgiveness. *J. Appl. Soc. Psychol.* 36 2362–2378. 10.1111/j.0021-9029.2006.00107.x

[B60] Takaku S. Weiner B. Ohbuchi K.-I. (2001). A cross-cultural examination of the effects of apology and perspective taking on forgiveness. *J. Lang. Soc. Psychol.* 20 144–166. 10.1177/0261927x01020001007

[B61] Thoresen C. E. Harris A. H. S. Luskin F. (2000). “Forgiveness and health: an unanswered question,” in *Forgiveness: Theory, Research, and Practice*, eds McCullough M. E. Pargament K. I. Thoresen C. E. (New York, NY: Guildford Press), 254–280.

[B62] Toussaint L. Shields G. S. Dorn G. Slavich G. M. (2016). Effects of lifetime stress exposure on mental and physical health in young adulthood: how stress degrades and forgiveness protects health. *J. Health Psychol.* 21 1004–1014. 10.1177/1359105314544132 25139892 PMC4363296

[B63] Tsang J. A. McCullough M. E. Fincham F. D. (2006). The longitudinal association between forgiveness and relationship closeness and commitment. *J. Soc. Clin. Psychol.* 25 448–472. 10.1521/jscp.2006.25.4.448

[B64] Wallace H. M. Exline J. J. Baumeister R. F. (2008). Interpersonal consequences of forgiveness: Does forgiveness deter or encourage repeat offenses? *J. Exp. Soc. Psychol.* 44 453–460. 10.1016/j.jesp.2007.02.012

[B65] Weiner B. (1985). An attributional theory of achievement motivation and emotion. *Psychol. Rev.* 92 548–573. 10.1037/0033-295X.92.4.548 3903815

[B66] Weiner B. (1995). *Judgments of Responsibility: A Foundation for a Theory of Social Conduct.* New York, NY: Guilford Press.

[B67] Weiner B. Graham S. Peter O. Zmuidinas M. (1991). Public confession and forgiveness. *J. Pers.* 59 281–312. 10.1111/j.1467-6494.1991.tb00777.x 15681676

[B68] Wenzel M. Turner J. K. Okimoto T. G. (2010). Is forgiveness an outcome or initiator of sociocognitive processes? Rumination, empathy, and cognitive appraisals following a transgression. *Soc. Psychol. Pers. Sci.* 1 369–377. 10.1177/1948550610376598

[B69] Witvliet C. V. O. McCullough M. E. (2007). “Forgiveness and health: a review and theoretical exploration of emotion pathways,” in *Altruism and Health: Perspectives from Empirical Research*, ed. Post S. G. (New York, NY: Oxford University Press), 259–276.

[B70] Witvliet C. V. O. Phipps K. A. Feldman M. E. Beckham J. C. (2004). Posttraumatic mental and physical health correlates of forgiveness and religious coping in military veterans. *J. Trauma Stress* 17 269–273. 10.1023/B:JOTS.0000029270.47848.e 15253099

[B71] Worthington E. L. Jr. (1998). An empathy-humility-commitment model of forgiveness applied within family dyads. *J. Fam. Ther.* 20 59–76. 10.1111/1467-6427.00068

[B72] Worthington E. L. Jr. (2001). *Five Steps to Forgiveness: The Art and Science of Forgiving.* New York, NY: Crown.

[B73] Worthington E. L. Jr. (2006). *Forgiveness and Reconciliation: Theory and Application.* New York, NY: Routledge.

[B74] Worthington E. L. Jr. Witvliet C. V. O. Lerner A. J. Scherer M. (2005). Forgiveness in health research and medical practice. *EXPLORE* 1 169–176. 10.1016/j.explore.2005.02.012 16781526

[B75] Worthington E. L. Jr. Witvliet C. V. O. Pietrini P. Miller A. J. (2007). Forgiveness, health, and well-being: a review of evidence for emotional versus decisional forgiveness, dispositional forgivingness, and reduced unforgiveness. *J. Behav. Med.* 30 291–302. 10.1007/s10865-007-9105-8 17453329

